# Deletion of Specific Conserved Motifs from the N-Terminal Domain of αB-Crystallin Results in the Activation of Chaperone Functions

**DOI:** 10.3390/ijms23031099

**Published:** 2022-01-20

**Authors:** Sundararajan Mahalingam, Goutham Shankar, Brian P. Mooney, Kamal Singh, Puttur Santhoshkumar, Krishna K. Sharma

**Affiliations:** 1 Department of Ophthalmology, School of Medicine, University of Missouri-Columbia, Columbia, MO 65212, USA; smmwt@missouri.edu (S.M.); gszcx@missouri.edu (G.S.); 2 Charles W. Gehrke Proteomics Center, Department of Biochemistry, University of Missouri-Columbia, Columbia, MO 65211, USA; mooneyb@missouri.edu; 3 The Christopher S. Bond Life Science Center, University of Missouri-Columbia, Columbia, MO 65211, USA; singhka@missouri.edu; 4 Department of Veterinary Pathobiology, University of Missouri-Columbia, Columbia, MO 65211, USA; 5 Department of Biochemistry, University of Missouri-Columbia, Columbia, MO 65211, USA

**Keywords:** αB-crystallin, chaperone, deletion mutant, oligomerization, structure, beta-amyloid, apoptosis, oxidative stress

## Abstract

Smaller oligomeric chaperones of α-crystallins (αA- and αB-) have received increasing attention due to their improved therapeutic potential in preventing protein aggregating diseases. Our previous study suggested that deleting 54–61 residues from the N-terminal domain (NTD) of αB-crystallin (αBΔ54–61) decreases the oligomer size and increases the chaperone function. Several studies have also suggested that NTD plays a significant role in protein oligomerization and chaperone function. The current study was undertaken to assess the effect of deleting conserved 21–28 residues from the activated αBΔ54–61 (to get αBΔ21–28, Δ54–61) on the structure–function of recombinant αBΔ21–28, Δ54–61. The αBΔ21–28, Δ54–61 mutant shows an 80% reduction in oligomer size and 3- to 25-fold increases in chaperone activity against model substrates when compared to αB-WT. Additionally, the αB∆21–28, ∆54–61 was found to prevent β-amyloid (Aβ_1–42_) fibril formation in vitro and suppressed Aβ_1–42_-induced cytotoxicity in ARPE-19 cells in a more effective manner than seen with αB-WT or αB∆54–61. Cytotoxicity and reactive oxygen species (ROS) detection studies with sodium iodate (SI) showed that the double mutant protein has higher anti-apoptotic and anti-oxidative activities than the wild-type or αB∆54–61 in oxidatively stressed cells. Our study shows that the residues 21–28 and 54–61 in αB-crystallin contribute to the oligomerization and modulate chaperone function. The deletion of conserved 21–28 residues further potentiates the activated αBΔ54–61. We propose that increased substrate affinity, altered subunit structure, and assembly leading to smaller oligomers could be the causative factors for the increased chaperone activity of αBΔ21–28, Δ54–61.

## 1. Introduction

Alpha B-crystallin is a member of the small heat-shock protein (sHsp) family that prevents misfolded target proteins from aggregation and precipitation [1,2,3]. α-crystallin’s chaperone activity can be compromised by mutation or posttranslational modifications [4], leading to protein aggregation and cataracts due to the insolubility of the mutant or modified proteins or due to the insolubility of the complexes formed between the gain of function mutants and cellular proteins [4,5]. Decades of in vitro studies have suggested that α-crystallins with chaperone-like activity may serve a protective function in the lens [1,6]. We previously created and characterized an activated form of recombinant αB-crystallin (αBΔ54–61) after deleting the 54–61 sequence [7]. The αBΔ54–61 mutant formed ~40% smaller oligomers compared to that of the wild-type protein, showed ~10-fold increase in chaperone-like function during alcohol dehydrogenase (ADH) aggregation assays [7] and superior anti-oxidative and anti-apoptotic activities compared to the wild-type protein [8] despite not having S59 phosphorylation site implicated in the modulation of chaperone-like activity. Phosphorylation of αB-crystallin is localized mainly to three S residues: S19, S45, and S59 [9,10]. Studies have shown that when these sites are phosphorylated, αB-crystallin forms smaller oligomeric complexes and shows preferential binding to different client proteins [11,12].

**Figure 1:**
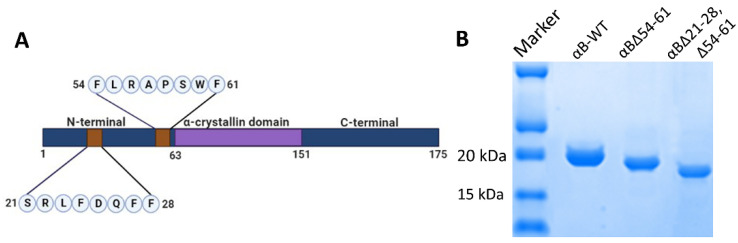
(**A**). Schematic representation of αB-crystallin showing central α-crystallin domain flanked by N-terminal domain and C-terminal domain and deleted 21–28 and 54–61 sequences. (**B**). SDS-PAGE profile of purified recombinant αB-WT, αBΔ54–61, and αBΔ21–28, Δ54–61 crystallins showing variability in monomeric size.

High-resolution structural studies on αB-crystallin suggest both N- and C-terminal extensions are essential for regulating oligomerization through domain swapping [13]. A few other studies have also indicated that the N-terminal region in sHsps play a role in oligomerization [7,14]. In an earlier study, Pasta et al. [15] showed that the deletion of the SRLFDQFFG sequence in αB-crystallin (αBΔ21–29) results in smaller oligomers with a significant increase in chaperone activity. We have observed similar effects with the αBΔ54–61 mutant. Therefore, the present study evaluated the impact of deletion of both SRLFDQFF and FLRAPSWF motifs (Figure 1A) on the structure and function of αB-crystallin and to see whether the mutant crystallin (αBΔ21–28, Δ54–61) can be exploited to protect cells from oxidative stress. There are no fully characterized double mutants of alpha B-crystallins that are functionally active. We aimed to create the most active form of αB-crystallin with great therapeutic potential.

Alzheimer’s disease (AD) and age-related macular degeneration (AMD) share several pathological features, including β-amyloid (Aβ) peptide accumulation, oxidative damage, and cell death [16]. Our long-term goal is to develop a crystallin protein that can diminish the toxicity of disease-causing proteins. Therefore, in this study, we have also investigated whether the toxic effect of β-amyloid can be mitigated by αBΔ21–28, Δ54–61-crystallin and if αBΔ21–28, Δ54–61 prevents β-amyloid fibril formation in vitro.

## 2. Results

### 2.1. Deleting 21–28 and 54–61 Residues from αB-Crystallin Results in Increased Chaperone Activity

**Figure 2:**
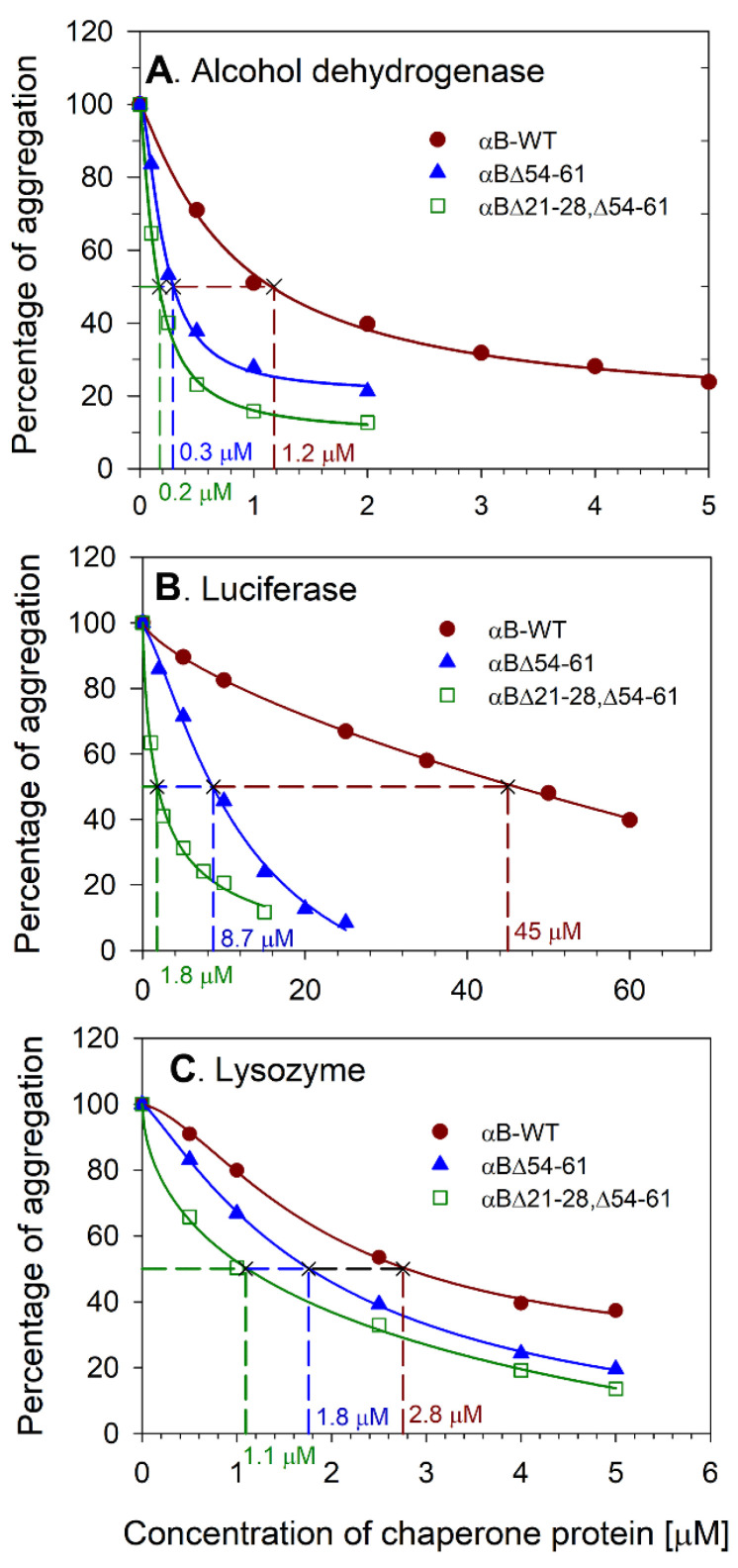
Comparison of anti-aggregation activities of αB∆21–28, ∆54–61, αB-WT, and αB∆54–61. Relative aggregation of (**A**) alcohol dehydrogenase, (**B**) luciferase, and (**C**) lysozyme in 0.25 mL assay buffer in the presence of different concentrations of chaperone proteins was estimated by monitoring the light scattering at 360 nm on a plate reader as described under methods. The result shown is representative of three independent experiments. The EC50 (effective chaperone protein concentration required to suppress the substrate protein aggregation by 50%) values are shown in the figure. The substrate protein aggregation (scattering at 360 nm) in the absence of chaperone protein is considered 100% aggregation. The aggregation of substrate protein shown at each concentration of chaperone protein tested is relative to the aggregation of substrate protein without the chaperone protein.

We investigated whether the removal of 21–28 residues from NTD of αBΔ54–61 affects the chaperone-like activity of double mutant (αBΔ21–28, Δ54–61) protein. The anti-aggregation activities of the WT and mutant chaperones were compared using three client proteins and are illustrated in Figure 2A–C. The αB-WT and deletion mutants showed a dose-dependent suppression of substrate proteins’ aggregation. With luciferase substrate, αBΔ21–28, Δ54–61 exhibited a 25-fold and 5-fold increase in chaperone activity compared with αB-WT and αBΔ54–61 respectively. The deletion mutants also showed increased anti-aggregation activity when alcohol dehydrogenase (ADH) or lysozyme were used as unfolding substrates. During the heat-induced ADH aggregation assay, the double mutant showed ~7-fold and a 2-fold increase in chaperone activities compared with αB-WT and αBΔ54–61, respectively. During chemical-induced aggregation of lysozyme, αBΔ21–28, Δ54–61 showed a 3-fold and 2-fold increase in chaperone activities compared to αB-WT and αBΔ54–61, respectively. The double deletion mutant showed superior anti-aggregation activity compared to wild-type and αBΔ54–61 proteins with all three substrates tested.

### 2.2. Deletion of 21–28 and 54–61 Residues in αB-Crystallin Leads to the Smaller Homo-Oligomers

**Figure 3:**
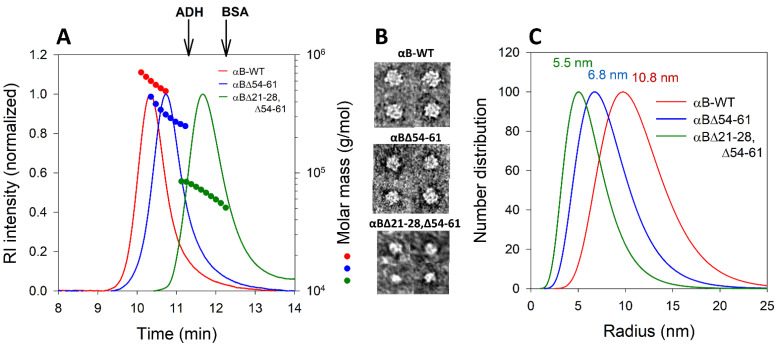
(**A**) Multi-angle light scattering analysis of αB-WT, αBΔ54−61, and αBΔ21–28, Δ54–61. Proteins (0.1 mg in 0.25 mL phosphate buffer) were incubated at 37 °C for one hour before analysis. Molar mass distribution across the refractive index (RI) peaks were analyzed using ASTRA software from the MALS data. (**B**) Negatively stained TEM micrograph particles of αB-WT, αBΔ54–61, and αBΔ21–28, Δ54–61 crystallins show the size and structural variability. (**C**) Hydrodynamic radii (Rh) were measured using 25 µM of protein in 0.25 mL PBS (pH 7.4) in a NanoBrook Omni in 8.5 mm UV micro cuvette (Brand Tech. Inc., Essex, CT, USA). The data shown is a representative profile of three independent analyses.

Our previously reported study [7] suggested that the deletion of 54–61 amino acid residues from the NTD of αB-WT protein led to ~40% reduction in the oligomeric mass of the protein. While the average molar mass of αB oligomers reduced from 558 kDa in αB-WT to 262 kDa in αBΔ54–61, in αBΔ21–28, Δ54–61 it was further decreased to 85 kDa (~80% reduction compared to αB-WT) in size. Multi-angle light scattering data clearly showed a significant difference in the oligomeric size of the three proteins analyzed (Figure 3A). The differences in the size of oligomers formed by αB-WT and two deletion mutants were also observed when negatively stained proteins were examined under transmission electron microscopy (TEM). The micrographs of particles of αB-WT (~12 nm), αBΔ54–61 (~8 nm), and αBΔ21–28, Δ54–61 (~6 nm) crystallins reflect the change in the size and structural variability of three proteins (Figure 3B). The particle size of the proteins analyzed using the NanoBrook Omni (Brookhaven Instruments, Holtsville, NY, USA) (Figure 3C) also supported the observations made with TEM. Furthermore, these results mirror the multi-angle light scattering profiles of all alpha B-crystallins studied.

### 2.3. Structural Characterization of the αB∆21–28, ∆54–61 Showed Significant Differences with αB-WT and αB∆54–61

**Figure 4:**
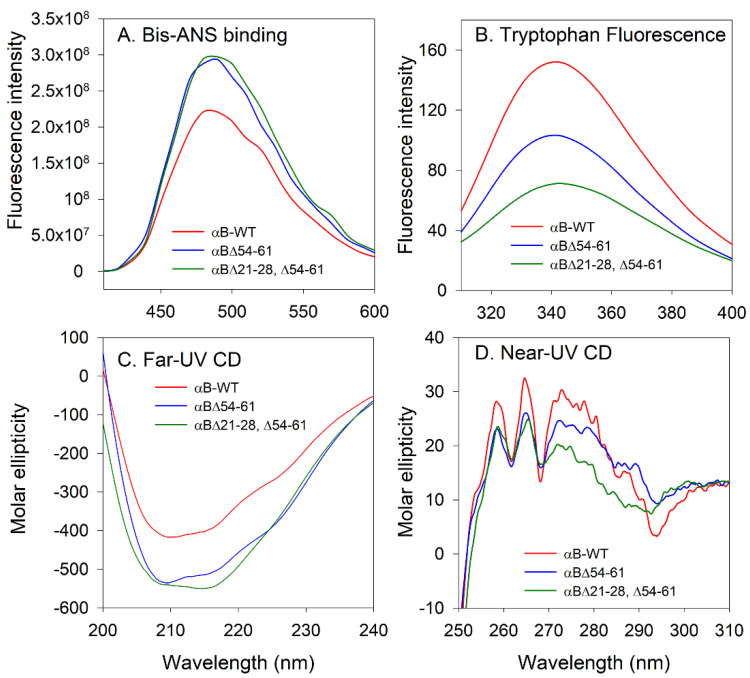
Spectroscopic characterization of αB-crystallins (**A**) Bis-ANS binding. Surface hydrophobicity measurements reflecting Bis-ANS binding to αB-crystallins (0.2 mg/mL in PBS pH 7.4) were recorded using 410 to 600 nm using 385 nm as the excitation wavelength. (**B**) Intrinsic tryptophan fluorescence of alpha B-crystallins (0.2 mg/mL in PBS pH 7.4) were recorded from 310 to 400 nm using 295 nm as the excitation wavelength. (**C**) Far-UV CD spectra were recorded at a 0.2 mg/mL protein concentration using a 2 mm path length cell. (**D**) Near-UV CD spectra were recorded at a 3 mg/mL protein concentration using a 5 mm path length cell.

The emission intensity of bis-ANS bound to αB∆21–28, ∆54–61, and αB∆54–61 showed no significant difference; however, a 26% increase in bis-ANS fluorescence was seen with the mutants when compared to αB-WT (Figure 4A). The mutants and wild-type αB-crystallins also showed significant differences in their intrinsic tryptophan emission spectra (Figure 4B). The maximal emission was found at 341 nm for both αB-WT and αB∆54–61. However, for αB∆21–28, ∆54–61 the maximum emission was found at 343 nm. The decrease in fluorescence intensity of the mutants suggests a quenching effect or an increased exposure of tryptophan residues to the solvent. The secondary and tertiary structures of αB∆21–28, ∆54–61 were investigated by far- and near-UV CD spectral analysis. The far-UV CD spectrum of the αB∆21–28, ∆54–61 crystallin exhibited maximal negative ellipticity at 214 nm, whereas for αB-WT and αB∆54–61 the minima were at 211 nm and 208 nm, respectively, indicating that all three proteins have a predominant β-sheet conformation at 25 °C (Figure 4C). The near-UV CD profile of the αB∆21–28, ∆54–61 showed characteristics such as those of the wild-type and αB∆54–61 proteins, except that the amplitude of the CD spectra between 275 to 290 nm was slightly lower (Figure 4D). The changes in the near-UV CD spectra are observed in the region contributed by tryptophan residues of αB∆21–28, ∆54–61 (Figure 4D). In total, the structural characterization studies suggest that αB∆21–28, ∆54–61 has a significantly altered structure compared to αB-WT.

### 2.4. αB∆21–28, ∆54–61 Is More Susceptible to Cleavage by Trypsin than the aB-WT

**Figure 5:**
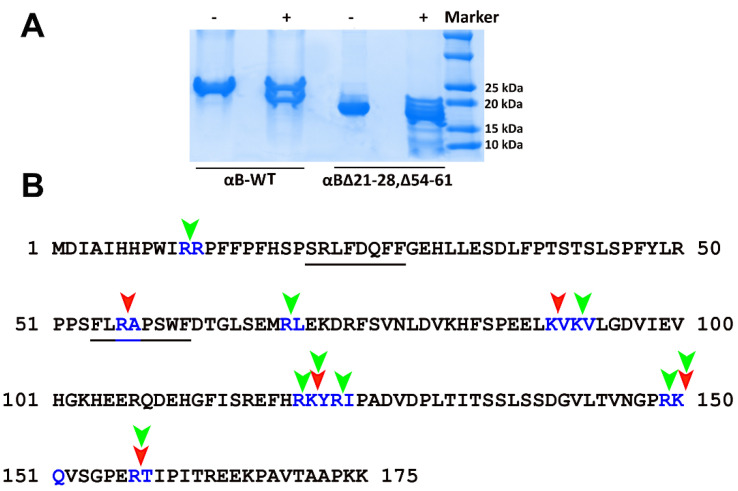
SDS-PAGE analysis of αB-WT and αB∆21–28, ∆54–61 after limited proteolysis using trypsin. (**A**). Twenty microgram aliquots of 1 mg/mL αB-WT and αB∆21–28, ∆54–61 were incubated with (+) or without (−) trypsin (200:0.5 protein to protease *w*/*w*) at 37 °C for 10 min. The reaction was terminated with the addition of trifluoroacetic acid to a final concentration of 0.1%. The extent of proteolysis was evaluated on a 4–20% SDS-PAGE gel. (**B**). Representation of tryptic cleavage sites in αB-WT and αB∆21–28, ∆54–61 observed from the samples digested for 10 min and subjected to LC-MS analysis. Arrow indicates the cleavage sites in the protein sequences (red = αB-WT; green = αB∆21–28, ∆54–61).

The limited proteolysis approach suggested that the deletion of 21–28 and 54–61 residues from the NTD results in conformational changes in the αB-crystallin oligomer, leading to the exposure of more tryptic sites that are otherwise masked in αB–WT oligomer. Multiple tryptic cleavage sites were observed in αB∆21–28, ∆54–61 after 10 min incubation with trypsin (Figure 5A), suggesting the exposure of tryptic cleavage sites in the double mutant. Further incubation of the protein with trypsin resulted in the complete hydrolysis of the protein at 30 min (data not shown). Comparing the mass spec data of trypsin-cleaved peptides generated from αB-WT and αBΔ21–28, Δ54–61 at ten mins showed that ^11^Arg-Arg^12^, ^69^Arg-Leu^70^, ^92^Lys-Val^93^, ^120^Arg-Lys^121^, ^123^Arg-Ile^124^, and ^149^Arg-Lys^150^ are the additional sites that became susceptible for trypsin action in the double mutant (Figure 5B; the targets for trypsin cleavage in the double mutant (green arrow) is shown corresponding to positions in the wild-type protein). The mass spectrometric results also indicated that the relative susceptibility of various cleavage sites varied. Further studies are required to qualitatively assess which peptide bonds become most susceptible to protease in the mutant protein compared to the wild-type crystallin.

### 2.5. αB∆21–28, ∆54–61 Shows Increased Anti-Amyloidogenic Potential In Vitro

**Figure 6:**
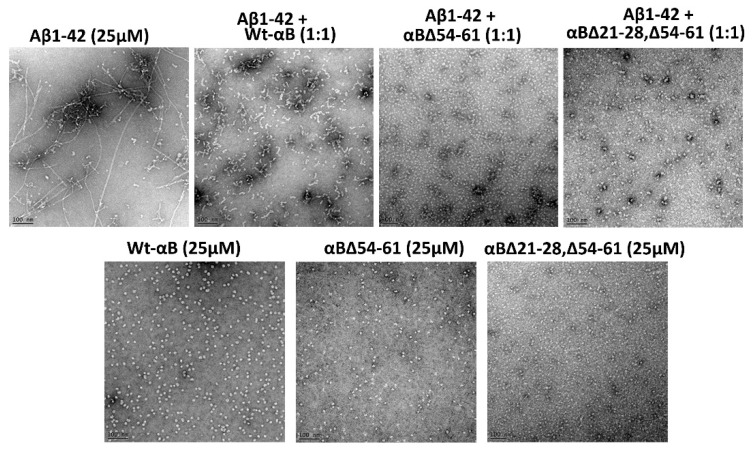
TEM micrographs of human Aβ_1–42_ with and without αBΔ21–28, Δ54–61 after 72 h of incubation in PBS buffer at 37 °C without shaking. Negatively stained (with 2% uranyl acetate) sample images were captured using a JEOL JEM 1400 electron microscope (JEOL Inc., Peabody, MA, USA). The scale bar is equal to 100 nm (25× magnification).

TEM images showed a distinct fibrillar structure when β-amyloid peptide alone was incubated for 72 h at 37 °C. The fibrils were long and mature in the β-amyloid peptide sample that was set for 72 h (Figure 6). However, when αB∆21–28, ∆54–61, or αB∆54–61 was co-incubated with β-amyloid at a 1:1 molar ratio, nearly complete suppression of fibril formation was observed (Figure 6). The double mutant protein by itself did not form amyloid fibrils under the experimental conditions used (Figure 6). The results strongly suggest that the double deletion mutant of alpha B-crystallin prevents the conversion of Aβ_1–42_ protofibrils into mature fibrils to a greater extent than by the WT- and αB∆54–61 proteins.

### 2.6. αBΔ21–28, Δ54–61 Crystallin Suppresses the Aβ_1–42_ Peptide-Induced Toxicity in ARPE-19 Cells

**Figure 7:**
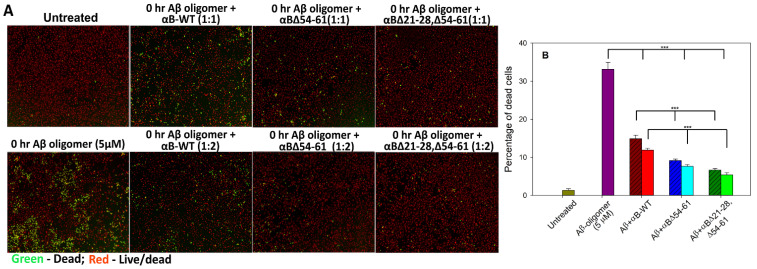
Suppression of Aβ_1–42_ peptide-induced cell death by αBΔ21–28, Δ54–61 crystallin. Panel (**A**) HFIP-treated Aβ_1–42_ (freshly prepared) and WT- and mutant αB-crystallins (1:1 and 1:2) were pre-incubated at 37 °C for 2 h and added to ARPE-19 cells cultured on a 96-well plate. After 24 h, the plate was stained with EarlyTox cell integrity kit (Molecular Devices, San Jose, CA, USA) and imaged as described under methods. Green = dead cells; Red = live/dead cells. Panel (**B**) Bar diagram showing the percentage of dead cells was calculated after live/dead cell imaging using SoftMax Pro software (Molecular Devices). The asterisks (***) indicate a *p*-value < 0.005 (*n* = 4). The patterned bars represent chaperone proteins when used at a 1:1 molar concentration of Aβ peptide. The plain bars in chaperone protein-treated samples represent Aβ and chaperone protein at 1:2 molar concentration.

It is well-known that Aβ_1–42_ peptide induces cell toxicity via oxidative stress [17]. To determine the effect of αBΔ21–28, Δ54–61 crystallin on Aβ_1–42_ peptide-induced cellular toxicity, we treated the ARPE-19 cells with hexafluoro-2-propanol (HFIP)-treated Aβ_1–42_ peptide and alpha B-crystallins in 1:1 and 1:2 molar ratios. Dead and live cells were stained with an EarlyTox cell integrity kit after 24 h. Aβ_1–42_ peptide-treated cells showed 33.14 ± 1.8% dead cells. In the presence of αB-WT (in 1:1 ratio), Aβ_1–42_-induced cell death was reduced to 15.00 ± 0.9%, whereas at 1:2 ratio of Aβ_1–42_ and αB-WT, the dead cells were 11.89 ± 0.4% (Figure 7). These values equal 55 and 64% protection of Aβ_1–42_-susceptible cells by αB-WT, respectively. In the presence of αB∆54–61 (1:1 ratio), Aβ_1–42_-induced cell death was reduced to 9.15 ± 0.3% (72% protection), and at twice the concentration of αB∆54–61, the cell death was reduced to 7.65 ± 0.4% (77% protection). Interestingly, cells simultaneously treated with 1:1 and 1:2 ratios of Aβ_1–42_ and αBΔ21–28, Δ54–61 showed 6.65 ± 0.41% and 5.41 ± 0.55% (equal to 80% and 84% protection, respectively) of cell death as shown in Figure 7, a significantly (*p* < 0.005) higher protection from cell death compared to αB-WT and αBΔ54–61. Overall data suggested that αBΔ21–28, Δ54–61 crystallin protected the Aβ_1–42_ peptide-induced toxicity more effectively than the αB-WT and αBΔ54–61 proteins. Results were consistent with three independent experiments and repeats.

### 2.7. αBΔ21–28, Δ54–61 Crystallin Blocks the Cytotoxic Action of Sodium Iodate and Increases Cell Viability on ARPE-19 Cells

**Figure 8:**
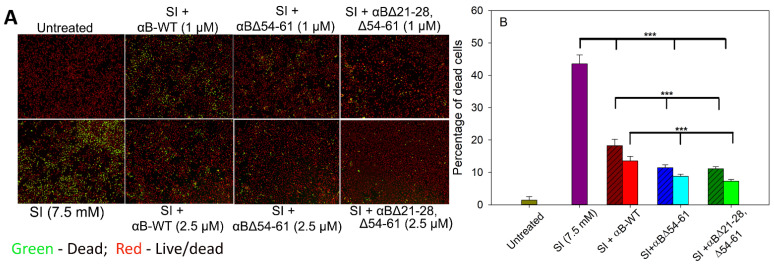
Cell integrity assay: (**A**)Effect of WT- and mutant αB-crystallins on SI-induced cytotoxicity in ARPE-19 cells. Serum-starved ARPE-19 cells were simultaneously treated with 1.0 or 2.5 μM proteins and 7.5 mM sodium iodate for 24 h. Cytotoxicity was measured using an EarlyTox cell integrity assay kit from Molecular Devices, San Jose, CA, USA. The cells were imaged on a SpectraMax MiniMax 300 Imaging Cytometer (Molecular Devices) equipped with a single 4× objective. (**B**) Bar diagram showing the percentage of dead cells calculated after live/dead cell imaging using SoftMax Pro software (Molecular Devices). The asterisks (***) indicate a *p*-value < 0.005 (*n* = 4). The patterned bars represent chaperone proteins when used at 1 µM. The plain bars in chaperone protein-treated samples represent chaperone protein when tested at 2.5 µM.

Sodium iodate (SI), a well-known chemical oxidant, has been shown to damage retinal pigment endothelial (RPE) cells [18,19]. In the present study, when ARPE-19 cells were treated with 7.5 mM SI for 24 h, 43.56 ± 3.2% of cells were found to be nonviable (Figure 8). Treating the cells simultaneously with 1 µM αB-WT reduced the dead cell population to 18.25 ± 1.9%, and at 2.5 µM of αB-WT, the SI-induced ARPE-19 cell death was further reduced to 13.51 ± 1.4% (*p* < 0.005). This activity equals 58% and 69% protection, respectively (Figure 8). The cells simultaneously treated with SI and 1 µM αBΔ54–61 showed 11.42 ± 0.89% cell death (74% protection), and at 2.5 µM αBΔ54–61, the cell death was reduced to 8.78 ± 0.69 (80% protection) (Figure 8). However, when 2.5 µM αBΔ21–28, Δ54–61 protein was added to cells in culture with SI, the cell protection increased significantly to 83%. At 1 µM, the protection offered by αBΔ21–28, Δ54–61 from SI-induced ARPE-19 cytotoxicity was similar to αBΔ54–61 when used at the same concentration (Figure 8). Therefore, the results strongly suggest that αBΔ21–28, Δ54–61 crystallin effectively blocks the cytotoxic actions of SI and increases ARPE-19 cell viability to a greater extent than wild-type and αBΔ54–61 proteins.

### 2.8. αBΔ21–28, Δ54–61 Attenuates Sodium Iodate-Induced Oxidative Stress in ARPE-19 Cells

**Figure 9:**
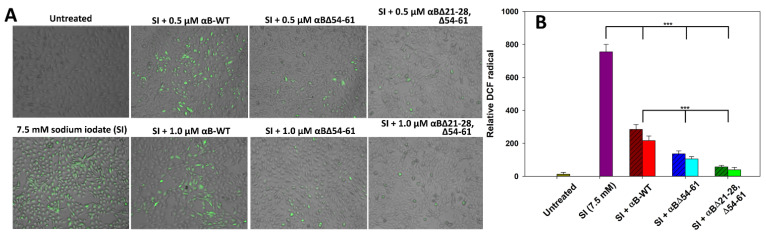
(**A**) Anti-oxidative potentials of αB∆21–28, ∆54–61 compared to αB-wt and αB∆54–61 in SI-treated ARPE-19 cells. ARPE-19 cells cultured on a 96-well plate were treated with SI and/or αB-crystallins for 24 h. The SI-induced reactive oxygen species (ROS) generation was measured by 2′,7′-Dichlorofluorescin diacetate (DCFH-DA) staining. The images were captured in EVOS FL Auto2 Imaging System (Thermo Fisher Scientific, Waltham, MA, USA) with 10× magnification. (**B**) The relative 2′,7′-dichlorofluorescein (DCF) intensity (green) was calculated using EVOS™ image analysis software (version 1.4.998.659). The data shown are an average of six analyses performed on images captured from different wells. The asterisks (***) represent a *p*-value < 0.005. The patterned bars represent chaperone proteins when used at 0.5 µM. The plain bars in chaperone protein-treated samples represent chaperone protein when tested at 1.0 µM.

The available reports from the last two decades suggest that sodium iodate can promote ROS generation closely associated with oxidative stress. It is known that ROS induces apoptosis in a variety of cells [18,20,21] that includes ARPE-19. Therefore, in the present study, we assessed the effect of αBΔ21–28, Δ54–61 on SI-induced stress in ARPE-19 cells. The non-fluorescent dichlorofluorescein (DCFH) is converted to DCF and emits fluorescence upon cellular oxidation. Because various ROS can oxidize DCFH, the increase in intracellular DCF fluorescence reflects an overall oxygen species index in cells. We have found that SI alone-treated cells in the present study showed increased relative DCF green fluorescence, indicating that treatment with SI can generate ROS in ARPE-19 cells (Figure 9).

However, we observed that treatment of SI-challenged cells with 0.5 and 1.0 µM WT-αB decreases ROS production by 62 ± 1.4% and 71 ± 0.9%, respectively. Under similar experimental conditions (Figure 9), ROS production in SI-treated cells reduced by 82 ± 1.1% and 86 ± 0.8%, respectively, when 0.5 and 1.0 µM αB∆54–61-crystallin was included in the culture (Figure 9). In contrast, SI-challenged cells treated with αB∆21–28, ∆54–61 at both concentrations showed a decrease in ROS production by 93 ± 0.5% and 95 ± 0.4%, respectively (Figure 9). These results indicate that SI-induced ROS in cells can be suppressed with αB∆21–28, ∆54–61, and the double deletion mutant has enhanced ROS blocking activity.

## 3. Discussion

The alpha-crystallins of the eye lens have become increasingly recognized for their role in maintaining lenticular transparency. Assessing the functional properties of αB, as monomer or oligomer, is essential to understanding its mode(s) of action. Furthermore, to produce the most active form of the protein for therapeutic use, necessary structural alterations (addition or deletion or substitution) in the amino acid sequence of the protein are also essential. Thus, the present study was undertaken to determine whether the removal of conserved amino acid residues 21–28 from the activated αB∆54–61 [7] will alter the structure and enhance the functional properties of the protein in vitro and its ability to protect cells under oxidative stress conditions.

Models of αB reported thus far have organized the protein in three domains: an NTD (60 residues), a central alpha-crystallin domain (ACD) (90 residues), and a C-terminal domain (CTD) of 25 residues containing the IXI motif. The NMR studies-based model reported by Jehle et al. [13] suggests the involvement of four regions of alpha B-crystallin in its multimerization. In addition, it has been shown that the ACD is involved in dimer formation, and the CTDs are responsible for defining hexameric units. Both NTD and ACD are responsible for higher-order multimerization [13]. The regions involved in one of the multimerization models are Leu37-Pro58, Phe75-Lys82 (β3), Leu131-Ser139 (β8), Gly141-Pro148 (β9), and Pro158-Glu165 (consisting of the IXI motif) [22].

We carried out the chaperone activity assays of the purified deletion mutants of αB-crystallin with different substrate proteins (Figure 2A–C) to determine the impact of the deletion of conserved [15] and subunit interaction regions [22] of the protein. The results show that the deletion of 21–28 and 54–61 residues increases the chaperone activity with different client proteins. With luciferase as substrate, αB∆21–28, ∆54–61 showed a marked increase in the chaperone activity compared to αB∆54–61 and αB-WT, as illustrated in Figure 2B. In an earlier study, we reported that αB∆54–61 has significantly higher chaperone activity than αB-WT [7,8]. Oligomer analysis showed that αB-WT is the largest oligomer, whereas αB∆21–28, ∆54–61 is the smallest and αB∆54–61 is of intermediate size (Figure 3A). A simplistic correlation of oligomeric size and chaperone efficiency of the three proteins, αB-WT and the two deletion mutants, suggests that the larger the oligomer is, the lower the chaperone efficiency. This could be due to the increased activity of smaller oligomeric species having a higher number of accessible binding sites per unit compared to the larger oligomers. This view is consistent with the observation made by Mühlhofer et al. [12]. Alternately, it is plausible that smaller oligomers have activated chaperone sites that are otherwise constrained by specific sequences in the NTD, as discussed earlier with αB∆54–61 that showed increased chaperone activity compared to αB-WT [8]. In various studies, single or double mutants of alpha-crystallins have also exhibited higher anti-aggregative effects [7,8,23,24], but none showed the activity comparable to that of αB∆21–28, ∆54–61.

The average molecular mass of the αBΔ21–28, Δ54–61 and full-length αB-WT determined by multi-angle light scattering suggests that the double mutant does not form large oligomers (Figure 3A–C). It has been reported that wild-type αB-crystallin forms oligomers of 500 to 650 kDa and that the N-terminal region plays a role in the oligomerization [7,15,25]. Since the deletion of a specific sequence from NTD leads to smaller oligomers, the N-terminal domain of αB-crystallin seems to be driving oligomer formation and dictating the size of the oligomers formed. The results of this study are consistent with the previous report, as the truncation of the N-terminal domain of αB-crystallin inhibited the formation of oligomers [26,27]. The average number of subunits in αB∆21–28, ∆54–61 oligomers is close to four. This is much lower than the estimated average subunits of 31 in αB-WT or 24 in αB∆54–61 [7]. It is unclear what factor(s) limit(s) the number of subunits in the oligomers formed by the single and double mutants of αB-crystallins or which factors dictate the oligomer size.

The secondary structure of the αB∆21–28, ∆54–61 protein showed predominant β-sheet conformation (Figure 4C). Deletion of 21–28 and 54–61 residues significantly affects the structure of the protein. The tryptophan fluorescence intensity was reduced by 32% in the αBΔ54–61 mutant compared with the wild-type protein, whereas in αB∆21–28, ∆54–61 it was reduced to 53%. However, the surface hydrophobicity of αB∆21–28, ∆54–61 appeared similar to that of αB∆54–61 (Figure 4A). The tryptophan fluorescence of αB∆21–28, ∆54–61 was likely reduced due to the deletion of the highly conserved residues from the NTD and deletion-induced changes in the subunit interactions. It is likely that the intrinsic fluorescence of lone Trp (residue 9) was reduced in αB∆21–28, ∆54–61 compared to that in αB∆54–61 due to the quenching brought about by the neighboring Tyr or His. It is known that Tyr and His quench Trp fluorescence [28]. The deletion of the 21–28 region also brings the His31 closer to Trp9, which is already in the vicinity of His 6, 7, and 17. Earlier, we observed that in αB-crystallin, intrinsic Trp fluorescence is significantly affected when 54–60 is inverted [29]. The inversion of 54–60 sequence in alpha B-crystallins resulted in a significant reduction in the intrinsic fluorescence of tryptophan compared to that of αB-WT crystallin. However, a decrease in the level of tryptophan fluorescence in the αB∆21–28, ∆54–61 had no bearing on chaperone activity, as reported above (Figure 2).

The deletion of αB∆21–28, ∆54–61 sequence also decreased the resistance of the protein to trypsin digestion compared to the αB-WT, suggesting that the smaller oligomers have exposed trypsin cleavage sites. The results of limited proteolysis and subsequent MS analysis revealed that the double mutant protein was more susceptible to trypsin. This could be due to the structural changes in the protein subunit and oligomer organization that exposes Arg-X or Lys-X bonds to trypsin. Earlier, we observed that the 54–61 region deletion from αB-crystallin also makes it more susceptible to trypsin than wild-type protein [8].

Amyloid fibrils accumulate and deposit in extracellular plaques, a hallmark of AD. The relative toxicity of fibrillar versus oligomeric amyloid is an area of controversy [30]. The size of the soluble amyloid (dimers, trimers, oligomers, or fibrils) that is most damaging is an area of active investigation [31]. Research suggests that each species of aggregates have a different degree of toxicity. However, a growing consensus suggests that amyloid oligomers have significantly higher toxicity than amyloid multimers [32]. To investigate which stage of the Aβ oligomer is showing toxicity to the cells, Aβ oligomers were prepared by incubating Aβ at 37 °C for 0 to 72 h and tested with ARPE-19 cells for 24 to 48 h. Cell viability assay showed that Aβ oligomer(s) formed immediately after dissolving with HFIP and added to ARPE-19 cells and cultured for 24 h showed the maximal cell toxicity (data not shown). Therefore, subsequent studies were carried out with these Aβ oligomers, and cell culture studies were continued for 24 h.

It has been reported that Aβ interaction with mitochondria may lead to increased free radical generation and subsequent cell death despite the mitochondrial anti-oxidant defense system [33,34]. We examined the ability of αB∆21–28, ∆54–61 to protect ARPE-19 cells from Aβ1-42 cytotoxicity because of the anti-amyloidogenic behavior of three αB-crystallins (Figure 6). αB∆21–28, ∆54–61 that forms smaller oligomers was found to have the highest anti-amyloid activity, including inhibition of Aβ1-42-induced cytotoxicity on ARPE-19 cells compared to αB-WT and αB∆54–61 (Figure 6 and Figure 7). Additional studies are needed to determine the molecular pathways involved in protecting ARPE-19 cells by αB∆21–28, ∆54–61.

Oxidative stress is one of the many factors in the pathogenesis of AMD and cataracts [35,36,37]. Oxidative stress can increase the level of ROS [38], deplete ATP [39], and promote plasma membrane leakage [40], DNA damage [41], and premature aging [41] in RPE cells. Exposure of RPE cells to SI has been shown to induce a loss of their integrity [42]. We investigated the cytoprotective effects of αB∆21–28, ∆54–61 on ARPE cells by employing SI-induced reactive oxygen species generation and apoptosis assay. During the study, we observed that the amount of ROS generated by SI was significantly reduced by the presence of αB∆21–28, ∆54–61. There were fewer ROS-positive cells, as shown by the DCF fluorescent intensity staining in the ARPE-19 cells (Figure 9). Our results strongly suggest that αB∆21–28, ∆54–61 protein effectively suppresses the cytotoxic action of sodium iodate compared to the efficacy of αB-WT and αB∆54–61 proteins.

SI has also been shown to be toxic to cells and causes the death of retinal cells in many mammals [43,44]. In the present study, SI at 7.5 mM showed toxic effects on the cells after 24 h (Figure 8). However, the cells co-treated with αB∆21–28, ∆54–61 showed a significant reduction in SI toxicity, evidenced by a greater degree of survival of cells (Figure 8). Furthermore, the activity of αB∆21–28, ∆54–61 was superior to that of αB-WT or αB∆54–61.

In conclusion, the current study results strongly support the hypothesis that the αB∆21–28, ∆54–61 possesses a novel paradigm with enhanced chaperone activity with a reduction in the oligomeric size and lacking phosphorylation site S59. The αB∆21–28, ∆54–61’s ability to inhibit SI-induced oxidative damage and maintain cell integrity, the novel features of the protein, makes it a potential candidate for further studies to treat protein aggregating diseases.

## 4. Materials and Methods

### 4.1. Construction of Plasmid DNA Expressing αB∆21–28, ∆54–61

Gene-expressing human αB∆21–28, ∆54–61 (without the C-terminal His-tag) and cloned onto pET23a (+) plasmid was obtained from GenScript USA Inc., Piscataway, NJ, USA. The in-frame T7 tag was removed by PCR using QuikChange II Site-Directed Mutagenesis Kit (Agilent Technologies, Santa Clara, CA, USA). The reading frame and the mutations were confirmed by DNA sequencing. The Plasmid DNA was transformed into *Escherichia coli* BL21(DE3) pLysS cells (Invitrogen Corp., Carlsbad, CA, USA) for expressing the mutant protein.

### 4.2. Overexpression and Purification of Wild-Type and αB-Crystallin Mutants

The wild-type, αB∆54–61, and αB∆21–28, ∆54–61 crystallins were expressed and purified as previously described [7]. In brief, the proteins were expressed in *Escherichia coli* BL21(DE3) pLysS cells (Invitrogen Corp., Carlsbad, CA, USA) with IPTG (0.5 mM) induction. The cells (from 1 L culture) were lysed in 15 mL of lysis buffer (50 mM Tris-HCl, 2 mM EDTA, 0.1 M NaCl (pH 7.5)) containing 50 µL of protease inhibitor cocktail III (Calbiochem-EMD Millipore Corporation, Billerica, MA, USA), lysozyme (0.1 mg/mL) (Worthington, Lakewood, NJ, USA) and benzonase nuclease (25 units) (Sigma-Aldrich, St. Louis, MO, USA). The lysates were centrifuged at 18,000× *g* for one hour, and the supernatants were collected. The αB-crystallin or its mutant was precipitated by ammonium sulfate (45%) treatment and collected by centrifugation. The protein precipitate was resolubilized in 3 mL of PBS (phosphate buffer saline) and purified by successive gel filtration chromatography on a HiLoad 16/60 Superdex G200 column (GE Healthcare Biosciences Corp, Piscataway, NJ, USA). The fractions containing the purified crystallins were pooled, concentrated, and stored as 3 mg/mL aliquots at −80 °C for further use. The Bio-Rad protein assay method was used to estimate the protein concentration. All protein concentrations shown are equivalent to monomeric concentrations. The purity of the samples was evaluated by SDS-PAGE and was over 95% (Figure 1B).

### 4.3. Chaperone Assays

The chaperone activities of all three αB-crystallins (WT- and deletion mutants) were measured using ADH (Worthington, Lakewood, NJ, USA), lysozyme, and luciferase (Promega, Madison, WI, USA) as aggregating substrates. The assays were carried out on a SpectraMax i3 plate reader (Molecular Devices, San Jose, CA, USA). The light scattering at 360 nm was recorded as a function of time in the presence or absence of αB-WT or αB∆54–61 or αB∆21–28, ∆54–61. ADH aggregation (100 µg) assay was performed at 37 °C in 0.25 mL of assay buffer (PBS containing 100 mM EDTA, pH 7.4) using 0–5 µM chaperone proteins. Aggregation assays for lysozyme (10 µM) were performed at 37 °C in 0.25 mL PBS. Two mM DTT (GoldBio, St Louis, MO, USA) was used to induce lysozyme aggregation, and 0–5 µM chaperone proteins were included in the assay. The luciferase (1 µM) aggregation was performed in PBS at 37 °C using 0–60 µM chaperone proteins. The percentage of substrate protein aggregated in the presence of various concentrations of the chaperone proteins at the end of the assay was calculated and plotted. The aggregation of the substrate protein in the absence of chaperone protein was considered 100% aggregation (control). The percentage of aggregation at a given concentration of the chaperone protein was calculated using the formula (absorbance of the sample/absorbance of the control) × 100. The relative chaperone efficiency of αB-WT, αB∆54–61, and αB∆21–28, ∆54–61 against a substrate was compared by estimating the EC50 (effective chaperone protein concentration required to suppress the substrate protein aggregation by 50%) values. The EC50 values were calculated from the non-linear regression analysis obtained by plotting the % of substrate protein aggregation at the end of the assay for a known chaperone protein concentration. Sigma plot (Version 12.5) (Systat Software Inc., Palo Alto, CA, USA) dynamic curve fitting with four-parameter logistic curve function was used for non-linear curve fitting analysis.

### 4.4. Structural Characterization of the αBΔ21–28, Δ54–61 Mutant

#### 4.4.1. Multi-Angle Light Scattering Analysis of αBΔ21–28, Δ54–61 Crystallin

Proteins (0.1 mg in 0.25 mL) were incubated at 37 °C for 1 h before analysis. The samples were injected into a TSK G5000PW_XL_ (Tosoh Bioscience, King of Prussia, PA, USA) size exclusion column attached to an HPLC with RID detector (Shimadzu Scientific Instruments, Inc., Columbia, MD, USA) and equilibrated with phosphate buffer. The flow rate was set at 0.75 mL/min. The HPLC eluant was fed to MALS (Wyatt Technology Corp., Santa Barbara, CA, USA). The molar mass distribution across the refractive index peaks was analyzed using ASTRA (6.1 v) software (Wyatt Technology, Santa Barbara, CA, USA) from the MALS data. The number of subunits per oligomer was estimated by dividing the oligomeric mass by the mass of the individual subunit.

#### 4.4.2. Transmission Electron Microscopy

TEM images of αB-WT, αBΔ54–61, and αBΔ21–28, Δ54–61 crystallin oligomers were collected in JEOL JEM 1400 transmission electron microscope after the proteins were stained with 2% uranyl acetate for 1 min on a 400-mesh carbon-coated copper grid.

#### 4.4.3. Fluorescence Measurements

Fluorescence spectra of proteins were recorded in a SpectraMax i3 plate reader (Molecular Devices, San Jose, CA, USA). Solvent-exposed surface hydrophobic sites in wild-type and mutant αB-crystallins were measured with protein hydrophobic site binding probe 1,1′-bi-(4-anilino) naphthalene-5,5′-disulfonic acid (Bis-ANS; Cayman Chemical, Ann Arbor, MI, USA). A stock solution of the Bis-ANS dye (14.8 mM) was prepared in 95% ethanol, and 200 µg/mL of crystallins were individually mixed with 10 µM of Bis-ANS after incubating at 37 °C for 15 min in the dark. The fluorescence spectra were recorded at 410 to 600 nm. The samples were excited at 385 nm. Three independent readings were collected to calculate the average.

The intrinsic tryptophan fluorescence spectra of the wild-type and two mutants of αB-crystallins were recorded using a Jasco FP-750 spectrofluorometer (Jasco Corporation, Tokyo, Japan), as described previously [7]. The excitation wavelength was set to 295 nm, and the emission was recorded between 310 and 380 nm. Protein samples of 200 µg/mL in phosphate buffer (pH 7.2) were used in spectral measurement studies.

#### 4.4.4. Far- and Near-UV CD Spectra

Mutation-induced changes in secondary and tertiary structures of crystallins were investigated by measuring far-ultraviolet (UV) and near-UV circular dichroism (CD) spectra using the Chirascan V100 Circular Dichroism Spectrometer (Applied Photophysics, Leatherhead, UK). Protein concentrations of 3.0 and 0.2 mg/mL in phosphate buffer (pH 7.2) were used for near- and far-UV CD measurements, respectively. The path length was 5 mm and 2 mm, respectively. The proteins were scanned five times, the spectra were averaged, and the molar ellipticity of the mutants was compared with that of wild-type αB-crystallin.

### 4.5. Limited Proteolysis of αB∆21–28, ∆54–61 Using Trypsin

Reconstituted αB-crystallins (WT- and double deletion mutant) were subjected to limited proteolysis to assess the effect of αB∆21–28, ∆54–61 on the susceptibility of oligomeric α-crystallin to the protease. αB-WT or αB∆21–28, ∆54–61 (200 µg) was individually mixed with 0.5 µg of sequencing-grade modified trypsin (Promega Corporation, Madison, WI, USA) at a ratio of 200:0.5 *w*/*w* in PBS pH 7.4, to a final reaction volume of 200 µL. From the parental mixture, 20 µg aliquots were incubated at 37 °C for 10 min. The reaction was terminated after 10 min using trifluoroacetic acid at a final concentration of 0.1%. Aliquots of samples (10 µg) were run on a 4–20% polyacrylamide gel and stained with Acqua protein gel stain (Bulldog Bio, Portsmouth, NH, USA). In parallel, the samples digested for 10 min were subjected to LC-MS analysis after diluting the peptide concentration to 1 pmol/µL. A portion of the sample (10 µL) was loaded onto a C8 trap column (Thermo Scientific Pepmap100, 300 µm × 5 mm, 5 µm C8). Bound peptides were eluted from this trap column onto a 10 cm × 150 µm i.d. pulled-needle analytical column packed with C8 reversed-phase resin (Michrom Bioresources, Auburn, CA, USA). Peptides were separated and eluted from the analytical column with a gradient of acetonitrile at 600 nL/min as follows: initial conditions 2% B (A: 0.1% formic acid in water; B: 99.9% acetonitrile, 0.1% formic acid), 2 min ramp to 20% B, gradient from 20–30% B over 15 min, ramp to 90% B over 5 min, hold at 90% B for 18 min, ramp back to (1 min) and hold at (4 min) initial conditions. The total run time was 45 min. The Proxeon Easy nLC HPLC system is attached to an LTQ Orbitrap XL mass spectrometer (Thermo Fisher Scientific, San Jose, CA, USA). High-resolution (60,000 resolution, 1 microscan, 5e5 AGC) FTMS data were acquired of the eluting fragments. In each cycle (approximately 3 s), the nine most abundant peptides were subjected to Ion-Trap CID MSMS (>2000 counts; 2 m/z mass window, normalized collision energy of 35%).

### 4.6. Anti-Amyloidogenic Potential of αB∆21–28, ∆54–61 Crystallin

The human Aβ1-42 peptide (Watson Bio, Houston, TX, USA) was pre-treated with hexafluoro-2-propanol (HFIP) solvent for 1 h at room temperature, sonicated, and completely dried using speed vac (Savant SC100 SpeedVac, Markham, ON, Canada) to maintain at unaggregated form as described by Stine et al. [45]. To examine the efficacy of the two mutants and WT protein in preventing the conversion of Aβ1-42 protofibrils into mature fibrils, 25 µM HFIP-treated Aβ1-42 peptide was incubated at 37 °C for 72 h in the presence and absence of alpha B-crystallins (αB-WT, αB∆54–61 and αB∆21–28, ∆54–61) in 1:1 and 1:2 molar ratios using phosphate buffer (pH 7.4) at the final volume of 100 µL. At the end of the incubation, tubes were centrifuged at 2000 rpm for 2 min, and a 5-μL sample from the bottom of the tube was placed on a 400-mesh carbon-coated copper grid for 30 s. Before placing the samples, the grids were glow discharged for 3 min using PELCO easiGlow™ glow discharge cleaning system (Ted Pella Inc., Redding, CA, USA). The excess solution was wicked off with blotting paper. The grid was washed three times with sterile filtered milli-Q water and stained for 1 min using 2% uranyl acetate. The negatively stained grid was air-dried and examined under JEOL JEM 1400 120 kV transmission electron microscopy (TEM). The TEM micrographs were captured on a digital camera at 25K magnification and processed using digital imaging software from Gatan Digital Micrograph (Gatan, Inc., Warrendale, PA, USA).

### 4.7. Suppression of β-Amyloid Cytotoxicity on ARPE-19 Cells by αB∆21–28, ∆54–61 Crystallin

Pre-authenticated ARPE-19 cells (ATCC, Manassas, VA, USA) were seeded in 96-well plates at 1.0 × 10^4^ cells (100 μL per well) in Dulbecco’s modified Eagle’s medium (DMEM/F12), containing 5% fetal bovine serum (FBS), 100 U/mL penicillin, and 100 µg/mL streptomycin at 37 °C in a humidified atmosphere of 5% CO_2_ and 95% air. When ARPE-19 cells’ confluency reached 60–70%, the cells were serum-starved for 2 h and cultured with 25 μM HFIP-treated Aβ_1–42_ (freshly prepared) with or without WT- or mutant αB-crystallins (1:1 and 1:2 molar ratio) pre-incubated at 37° C for 2 h. After 24 h, the cells were stained with an EarlyTox Cell Integrity assay kit (Molecular Devices, San Jose, CA, USA) to detect and differentiate between live and dead cells. The extent of dead cells was estimated by measuring the fluorescence intensity at 541 nm (green) in a Molecular Devices plate reader SpectraMax I3 (Molecular Devices, San Jose, CA, USA). The representative data shown are the average readings from four wells per sample. Data were analyzed by the ANOVA single factor test for statistical significance. All experiments were repeated three times.

### 4.8. Effect of αB∆21–28, ∆54–61 Crystallin on Sodium Iodate-Induced Oxidative Stress and Cytotoxicity on ARPE-19 Cells

Pre-authenticated ARPE-19 cells were cultured on a 96-well plate and simultaneously treated with 7.5 mM of sodium iodate and αB-crystallins (1 or 2.5 µM) in serum-free F-12/DMEM for 24 h. After incubation, the cells were stained with an EarlyTox cell integrity kit to detect and differentiate between live and dead cells. The data shown are an average of four analyses performed on images captured from different wells in a SpectraMax I3 (Molecular Devices LLC, San Jose, CA, USA).

### 4.9. Anti-Oxidant Action of αB∆21–28, ∆54–61 Crystallin on ARPE-19 Cells

To evaluate the ROS scavenging ability of αB-crystallins on ARPE-19 cells, 2′,7′-Dichlorofluorescin diacetate (DCFH-DA) assay was performed [8]. Following the cell growth conditions described above, ARPE-19 cells were seeded on a 96-well plate and simultaneously treated with 7.5 mM of sodium iodate and αB-crystallins (wild-type or mutants, 0.5 or 1.0 µM) using serum-free F-12/DMEM at the final volume of 100 μL for 24 h. After that time, the cells were stained with 10 μM DCFH-DA for 30 min at 37 °C in the dark as per the manufacturer’s protocol and were observed under the EVOS FL Auto2 imaging system (Thermo Fisher Scientific, Waltham, MA, USA) with 10× magnification.

### 4.10. Statistical Analysis

Differences between the groups were assessed using a one-way ANOVA single factor test for statistical significance using SPSS for Windows (version 21, IBM Corporation, New York, NY, USA) and SigmaPlot (version 12.5) graphics software (Systat Software Inc., Palo Alto, CA, USA). When one-way ANOVA yielded significant results, post hoc testing was performed for inter-group comparisons using the least significant difference test. Values corresponding to *p* < 0.05 were considered statistically significant and are denoted by distinct symbols in the tables and figures. The values are expressed as the mean ± standard deviation of multiple readings.

## Data Availability

None.
